# Chronic Pain and Its Association with Depressive Symptoms and Renal Function in Hypertensive Patients

**DOI:** 10.3390/ijerph19031899

**Published:** 2022-02-08

**Authors:** Rigas G. Kalaitzidis, Panagiotis Theofilis, Kalliopi Touchantzidou, Aikaterini Vordoni, Kostas C. Siamopoulos, Petros Skapinakis

**Affiliations:** 1 Center for Nephrology “G. Papadakis”, General Hospital of Nikaia—Piraeus “Agios Panteleimon”, 18454 Piraeus, Greece; panos.theofilis@hotmail.com (P.T.); katerinavord@gmail.com (A.V.); 2 2nd Cardiology Department, General Hospital of Nikaia—Piraeus “Agios Panteleimon”, 18454 Piraeus, Greece; kalliopitouch@gmail.com; 3 Nephrology Department, University of Ioannina School of Medicine, 45110 Ioannina, Greece; ksiamop@gmail.com; 4 Psychiatry Department, University of Ioannina School of Medicine, 45110 Ioannina, Greece; p.skapinakis@gmail.com

**Keywords:** chronic pain, hypertension, chronic kidney disease, depression

## Abstract

Chronic pain is a common concern and is considered to be one of the major problems in patients with chronic physical disorders. We studied the effect of pain in patients with hypertension with or without chronic kidney disease (CKD) and the association between pain and symptoms of depression. The study involved 158 hypertensive individuals (59.5% male, mean age 55 years), of whom 47 (29.8%) had CKD (estimated glomerular filtration rate < 60 mL/min/1.73 m^2^). Pain was assessed with the pain/discomfort domain of the EuroQol-5 D, while depressive symptoms were assessed with the depression module of the Patient health questionnaire (PHQ-9). The prevalence of chronic pain in our sample was 44.3%. Women exhibited chronic pain more often compared to men (57.1% vs. 42.9%, *p* < 0.001). The presence of CKD was not significantly associated with a higher prevalence of chronic pain among hypertensive patients. Depressive symptoms were significantly associated with the presence of chronic pain. These findings were confirmed in the logistic regression analysis. Chronic pain is common in hypertensive individuals and the association with depression warrants further investigation and may have practical implications in managing these patients.

## 1. Introduction

Chronic pain is considered to be one of the biggest health problems globally since several million people suffer daily from ineffective control of their pain [1]. Chronic pain is often associated with common mental disorders such as anxiety and depression, further affecting the quality of life of these patients [2].

Arterial hypertension is an increasingly common noncommunicable disease that has been associated with chronic pain and depressive disorders. The prevalence of hypertension in the USA in 2017–2018 was 49.64%, corresponding to 115 million persons. The overall rate of well-controlled hypertension was 39.64%. Significant discrepancies exist in the burden and control rates in different subpopulation categories [3]. The interrelationship between elevated blood pressure (BP) and kidney disease has been well-established [4], and it is, therefore, unsurprising that chronic kidney disease (CKD) is a frequently encountered entity. Specifically, the adjusted prevalence of CKD (stages 1–5) varied between 3.31% in Norway and 17.3% in northeast Germany [5]. Additionally, the prevalence of CKD is 71.9 per 1000 individuals in the Canadian population [6]. Moreover, CKD is associated with increased cardiovascular morbidity and mortality, with higher prevalence in developing countries [7].

On the other hand, chronic pain is a prevalent symptom in patients with CKD due to CKD complications [8]. The control and the investigation of chronic pain can help understand the role of medication and provide safe care to people with CKD [9]. However, there are not enough data concerning the epidemiology of chronic pain in hypertensive patients with CKD. The purpose of this study was to assess (a) the prevalence of chronic pain in hypertensive patients with or without renal dysfunction and (b) to investigate the association of chronic pain with depression in CKD patients.

## 2. Materials and Methods

### 2.1. Study Design

This study was conducted with a cross-sectional design, that was chosen to allow us to examine associations between multiple variables of interest and pain in our study population. Individuals with chronic arterial hypertension who visited the outpatient department of the University Hospital of Ioannina as a part of their scheduled follow-up were enrolled. Their prior arterial hypertension diagnosis was based on the European Society of Cardiology/European Society of Hypertension guidelines, with confirmation by repeated office visits or ambulatory/home blood pressure measurements [10]. Exclusion criteria consisted of (a) history of cancer, (b) history of autoimmune disease, (c) history of arthritis, (d) acute illness. Following an interview, the data regarding patients’ sociodemographic characteristics and medical history (age, sex, history of diabetes mellitus [DM], and cardiovascular disease [CVD]) were collected. Body mass index (BMI) was calculated according to standard procedures and obesity was present in case of BMI ≥ 30 kg/m^2^. Resting arterial BP measurement was performed three times at 1–2 min intervals in the right arm by an automated sphygmomanometer, with the individual in a sitting position after 5 min rest. The recorded BP was the average of the last two readings. Pulse pressure was estimated according to the following formula: Pulse pressure = systolic BP − diastolic BP [11].

All individuals were informed about the study’s aims and provided written informed consent. The study was approved by the hospitals’ ethics committees (protocol number: 35443/18-12-2013) and was carried out according to the Declaration of Helsinki (1989).

### 2.2. Assessment of Chronic Pain

Symptoms of pain were assessed using the EuroQol (EQ)-5 D health self-assessment questionnaire. This questionnaire has been used previously in the quality-of-life assessment of hypertensive and chronic kidney disease patient populations [12,13,14,15]. In this study, we used data from the pain/discomfort domain based on the question that asked subjects to rate “pain/discomfort” as either “I have no pain or discomfort”, “I have moderate pain or discomfort”, or “I have extreme pain or discomfort”. Participants reporting the existence of at least moderate pain/discomfort were specifically asked about the duration of symptoms, with a duration of over three months defining chronicity. Based on these questions, we classified patients into two categories (as either reporting chronic pain or not).

### 2.3. Assessment of Depressive Symptoms

We used the depression module of the patient health questionnaire (PHQ-9) to assess depressive symptoms [16]. This is a short questionnaire assessing the nine symptoms of depression included in the DSM-V diagnostic criteria during the past 14 days. Scores for each item range from 0 (not at all) to 3 (nearly every day). Total scores range from 0 to 27. No information was collected for the duration of symptoms or previous history of depression. This questionnaire has been used in several studies in primary care and has been extensively used in Greece as well [17,18,19].

### 2.4. Laboratory Measurements

Individuals were assessed regarding hematologic parameters (hemoglobin, hematocrit, and fibrinogen) and biochemical parameters, including serum glucose, serum calcium, serum albumin, and C-reactive protein (CRP). A standard lipid panel was ordered, consisting of total cholesterol, high-density lipoprotein cholesterol (HDL-C), and triglycerides. The low-density lipoprotein cholesterol (LDL-C) concentration was estimated via the Friedewald formula [20]. The serum urea and creatinine measurement were also performed, and the estimated glomerular filtration rate was calculated according to the CKD-EPI equation [21]. Patients were additionally classified according to the presence of impaired renal function (eGFRCKD-EPI < 60 mL/min/1.73 m^2^).

### 2.5. Statistical Analysis

Continuous variables were assessed for normality of distribution with the Kolmogorov–Smirnov test and the visual inspection of P-P plots, and are presented as mean and standard deviation (SD). Categorical values are presented as percentages. The Student’s *t*-test was used to assess differences of continuous variables between the study groups, while the percentage differences were estimated by the formation of contingency tables and the performance of the chi-square test. A logistic regression analysis was performed to assess further the associations between self-reported chronic pain and its predictors, as these were detected from univariate tests. All reported *p*-values were based on two-sided hypotheses, with a *p*-value of 0.05 being considered statistically significant. All statistical calculations were performed using SPSS software (version 25.0; SPSS Inc., Chicago, IL, USA).

## 3. Results

### 3.1. Baseline Characteristics of the Study Population

A total of 158 patients were included in our study, and their demographic, clinical, and psychometric characteristics according to eGFRCKD-EPI are displayed in Table 1. Compared to patients with normal renal function, patients with impaired renal function were slightly older (58.3(SD: 13.8) years vs. 53.8(SD: 13.4) years, *p* = 0.07) and had similar BMI (28.1 (3.9) kg/m^2^ vs. 29.3(5.1) kg/m^2^, *p* = 0.22), systolic arterial pressure (130 (16) mmHg vs. 131(15) mmHg, *p* = 0.62), diastolic arterial pressure (86 (9) mmHg vs. 87 (10) mmHg, *p* = 0.68), and pulse pressure (44 (13) mmHg vs. 44 (12) mmHg, *p* = 0.79). In addition, there were no differences in the history of CVD (9.8% vs. 6.7%, *p* = 0.55), DM (19.5% vs. 10.1%, *p* = 0.14), and obesity (31.4% vs. 37.0%, *p* = 0.23) between the 2 groups (Table 1). Regarding the depressive symptoms, patients with impaired renal function had higher PHQ-9 scores (eGFR < 60: 6.5 (5.2) vs. eGFR ≥ 60: 4.7 (4.0), *p* = 0.02).

### 3.2. Laboratory Parameters of the Study Population

We additionally examined the association of several laboratory parameters with the presence of impaired renal function (Supplementary Table S1). Significant differences between the 2 groups were observed in the values of serum glucose levels (eGFR < 60: 116 (45) mg/dL vs. eGFR ≥ 60: 99 (17) mg/dL, *p* = 0.001), serum creatinine (eGFR < 60: 1.70 (0.61) mg/dL vs. eGFR ≥ 60: 1.03 (0.89) mg/dL, *p* < 0.001), urea (eGFR < 60: 61 (29) mg/dL vs. eGFR ≥ 60: 38 (21) mg/dL, *p* < 0.001), and the calculated glomerular filtration with the CKD-EPI type (eGFR < 60: 42.9 (11.0) mL/min/1.73 m^2^ vs. eGFR ≥ 60: 82.1 (13.7) mL/min/1.73 m^2^, *p* < 0.001), as expected. No major alterations were noted in the rest of the laboratory parameters assessed.

### 3.3. Determinants of Chronic Pain in Hypertension

**Figure 1:**
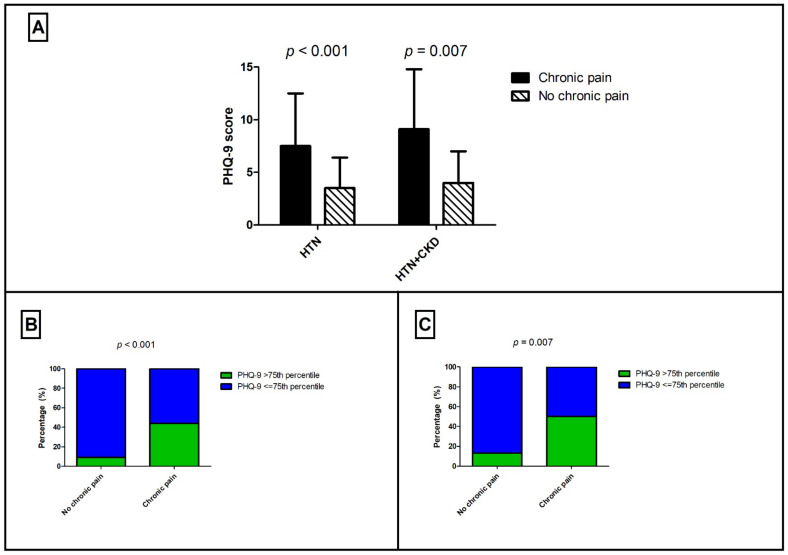
Association between depression and chronic pain. (**A**) Hypertensive patients with or without impaired renal function reporting chronic pain have higher scores of depression assessed by patient health questionnaire-9. The presence of PHQ-9 scores above the 75th percentile is associated with the presence of self-reported chronic pain in hypertensive patients (**B**) and in hypertensive patients with impaired renal function (**C**).

We next stratified our study population according to self-reported chronic pain (Table 2). The prevalence of chronic pain in our sample was 44.3%. Interestingly, patients with self-reported chronic pain were older (chronic pain: 58.7 (14.6) years vs. no chronic pain: 52.4 (12.3) years, *p* = 0.004), were more frequently female (chronic pain: 56.1% vs. no chronic pain: 26.3%, *p* < 0.001), and diabetic (chronic pain: 19.6% vs. no chronic pain: 8.1%, *p* = 0.05). Moreover, patients who reported chronic pain had higher scores on the PHQ-9 (chronic pain: 7.5 (5) vs. no chronic pain: 3.5 (2.9) years, *p* < 0.001) (Figure 1A) and a higher percentage of PHQ-9 scores above the 75th percentile (chronic pain: 44.1% vs. no chronic pain: 9.2%, *p* < 0.001) (Figure 1B). However, no significant differences were observed concerning BMI, eGFR, and CVD history. Furthermore, the presence of chronic pain was associated with lower hemoglobin (chronic pain: 13.6 (1.3) g/dL vs. no chronic pain: 14.3 (1.7) g/dL, *p* = 0.02), while hematocrit (chronic pain: 41.1 (3.6) % vs. no chronic pain: 42.3 (4.2) %, *p* = 0.08), and eGFR did not differ significantly (chronic pain: 67.0 (21.8) mL/min/1.73 m^2^ vs. no chronic pain: 73.1 (22.1) mL/min/1.73 m^2^, *p* = 0.09) (Supplementary Table S2).

To further assess the potential association of self-reported chronic pain with depression in hypertensive subjects, we performed a multivariable logistic regression analysis (Table 3), adjusting for important confounders. Even after adjustment for age, female sex, the presence of DM, hemoglobin level, and eGFR, patients with PHQ-9 scores above the 75th percentile were more likely to report chronic pain compared to patients with lower scores on the depression scale (OR 4.91, confidence intervals: 1.30 to 18.49). Female sex also remained significantly associated with chronic pain after the regression analysis (OR 2.87, confidence intervals: 1.13 to 7.33).

### 3.4. Chronic Pain in Patients with Impaired Renal Function

Finally, we assessed patients with impaired renal function (eGFRCKD-EPI < 60 mL/min/1.73 m^2^) stratified by self-reported chronic pain (Supplementary Table S3). Patients with self-reported chronic pain were older (chronic pain: 63.4 (12.1) years vs. no chronic pain: 53.3 (13.9) years, *p* = 0.01), and were more frequently female (chronic pain: 60.9% vs. no chronic pain: 25%, *p* = 0.01). With regards to depression, patients with impaired renal function who reported chronic pain had higher scores on the depression scale (chronic pain: 9.1 (5.7) vs. no chronic pain: 4 (3), *p* < 0.001) (Figure 1A) and a greater percentage of PHQ-9 scores above the 75th percentile (chronic pain: 50% vs. no chronic pain: 13%, *p* = 0.007) (Figure 1C). As far as the association of chronic pain with laboratory parameters is concerned, we detected lower levels of hemoglobin (chronic pain: 13.3 (1.4) g/dL vs. no chronic pain: 14.5 (1.8) g/dL, *p* = 0.05) and serum albumin (chronic pain: 3.9 (0.7) g/dL vs. no chronic pain: 4.4 (0.4) g/dL, *p* = 0.02) in patients with impaired renal function reporting chronic pain.

## 4. Discussion

Arterial hypertension represents a highly prevalent entity that rarely appears in isolation and is frequently accompanied by several comorbidities, such as diabetes mellitus, obesity, coronary artery disease, and heart failure [22]. As a result, a multidisciplinary approach is usually warranted for the most appropriate multifactorial management of hypertensive patients [23]. However, certain aspects of hypertension management remain incompletely understood, including the relationship of hypertension with chronic pain. According to earlier studies with a sound pathophysiologic basis, chronic pain may be considered a risk factor for the incidence of hypertension [24], while a recent cross-sectional study has documented an association between chronic pain and hypertension [25].

Chronic pain was a very frequent characteristic of our hypertensive patient population, with a prevalence rate of 44%. In the logistic regression analysis, the female sex was independently associated with chronic pain while other established cardiovascular risk factors were not associated with chronic pain. Previous studies have shown that patients with chronic pain are more likely to be older and female [26], in accordance with our findings. Moreover, chronic pain in hypertensive individuals with impaired renal function (eGFRCKD-EPI < 60 mL/min/1.73 m^2^) was also highly prevalent at a rate of approximately 49%. Although its frequency was similar compared to those with preserved renal function (eGFRCKD-EPI ≥ 60 mL/min/1.73 m^2^), our results confirm previous data showing that chronic pain is a very common symptom in people with CKD [9,27,28,29]. The epidemiology of chronic pain has not been adequately assessed in non-dialysis dependent CKD, as our patient population, with a recent meta-analysis reporting a higher prevalence according to one study [29]. One possible explanation for this discrepancy with our study may be the low prevalence of chronic pain risk factors in our CKD patients [1]. The high prevalence of pain in this subgroup of patients implies a significant potential for pain management in routine clinical practice. Indeed, there is a growing international trend towards increasing efforts to integrate the principles of palliative care, pain, and symptom management into the care of patients with chronic kidney disease [30].

Besides the well-established comorbidities in hypertension, depression is commonly encountered in hypertensive patients, as reported in a recent systematic review and meta-analysis [31]. Depression has also been associated with inadequate BP control [32], while improvement of depressive symptoms might result in BP lowering [33]. Chronic pain is an additional consideration in the interplay of hypertension with depression since the role of chronic pain and depression in BP-lowering treatment efficacy and adherence has been recognized [34]. In our study, hypertensive participants with depressive symptoms in the upper quartile (over 75th percentile) experienced chronic pain at a significantly higher rate compared to those who did not have depressive symptoms, a finding that was also present in the subgroup of patients with impaired renal function. The coexistence of chronic pain and depression is a challenging combination for managing patients in primary health care. It has been reported that 35% of patients with chronic pain have depression as comorbidity [35]. Most importantly, depression remained as an independent predictor of pain in our hypertensive cohort after the logistic regression analysis, highlighting its potential importance as a risk factor.

Our study has inherent limitations. To begin with, the associations observed cannot imply causality due to the cross-sectional design of our study. It should also be noted that none of the patients had end-stage renal disease, which may be related to a higher prevalence of chronic pain and depressive disorders [36,37], mandating further research in that field as these factors may hinder the quality of life of affected patients [37,38]. Moreover, the small number of individuals recruited might be insufficient to constitute potent associations. Furthermore, causes of chronic pain due to other conditions could not be assessed, and it is uncertain if they would have accounted for residual confounding. Last but not least, the presence of pain was based on a simple self-reported questionnaire and not according to other more detailed measures.

## 5. Conclusions

We found that hypertensive individuals report chronic pain at high rates. A significantly higher prevalence of chronic pain was detected in patients with depression scores at the highest percentile than those who do not have depression. Based on the potential causal association between chronic pain and arterial hypertension as well as the association of pain with depressive symptoms in our hypertensive cohort, the prompt recognition and management of pain and depression appears reasonable in order to achieve BP goals and reduce the excess morbidity and mortality associated with poor BP control. Future studies are needed to validate our findings and prospectively assess the importance of interventions towards pain and depression in hypertensive patients.

## Figures and Tables

**Table 1:** Demographic, clinical, and psychometric characteristics of the study population stratified by estimated glomerular filtration rate (eGFR).

Parameters	Total Patients (*N* = 158)	Patients with eGFR < 60 (*N* = 47)	Patients with eGFR ≥ 60 (*N* = 111)	*p*
**Sociodemographic and Clinical characteristics**				
Age, years	55.2 (SD: 13.7)	58.3 (SD: 13.8)	53.8 (SD: 13.4)	0.07
Male Sex	94 (59.5%)	27 (57.4%)	67 (60.4%)	0.73
BMI, kg/m^2^	28.9 (SD: 4.8)	28.1 (SD: 3.9)	29.3 (SD: 5.1)	0.22
Obesity	41 (35.3%)	11 (31.4%)	30 (37.0%)	0.23
Diabetes mellitus	17 (13.1%)	8 (19.5%)	9 (10.1%)	0.14
Cardiovascular disease	10 (7.7%)	4 (9.8%)	6 (6.7%)	0.55
SBP, mmHg	131 (SD: 15)	130 (SD: 16)	131 (SD: 15)	0.62
DBP, mmHg	87 (SD: 10)	86 (SD: 9)	87 (SD: 10)	0.68
PP, mmHg	44 (SD: 12)	44 (SD: 13)	44 (SD: 12)	0.79
**Depressive Symptoms**				
Depression score (PHQ-9)	5.2 (SD: 4.4)	6.5 (SD: 5.2)	4.7 (SD: 4.0)	0.02
PHQ-9 above 75th percentile	38 (24.5%)	14 (31.1%)	24 (21.8%)	0.22

BMI—body mass index, SBP—systolic blood pressure, DBP—diastolic blood pressure, PP—pulse pressure, PHQ-9—patient health questionnaire-9.

**Table 2:** Clinical and psychometric characteristics of the study population stratified by self-reported pain (*N* = 158).

Parameters	Chronic Pain (*N* = 70)	No Chronic Pain (*N* = 88)	*p*
**Clinical characteristics**			
Age, years	58.7 (SD: 14.6)	52.4 (SD: 12.3)	0.004
Male Sex	30 (42.9%)	64 (72.7%)	<0.001
BMI, kg/m^2^	29.6 (SD: 5.3)	28.4 (SD: 4.4)	0.19
Obesity	21 (41.2%)	20 (30.8%)	0.24
Diabetes mellitus	11 (19.6%)	6 (8.1%)	0.053
eGFR < 60 mL/min/1.73 m^2^	23 (32.9%)	24 (27.3%)	0.45
Cardiovascular disease	3 (5.4%)	7 (9.5%)	0.39
**Psychometric characteristics**			
Depression (PHQ-9)	7.5 (SD: 5.0)	3.5 (SD: 2.9)	<0.001
PHQ-9 above 75th percentile	30 (44.1%)	8 (9.2%)	<0.001

BMI—body mass index, eGFR—estimated glomerular filtration rate, PHQ-9—patient health questionnaire-9.

**Table 3:** Logistic regression analysis: Associations of chronic pain in hypertensive patients.

Parameter	Odds Ratio	95% Confidence Interval	*p*
Age	1.03	0.99–1.07	0.12
Female sex	2.87	1.13–7.33	0.03
Diabetes mellitus	2.20	0.60–8.02	0.23
Hemoglobin	0.78	0.57–1.06	0.10
eGFR_CKD-EPI_	0.99	0.97–1.01	0.40
PHQ-9 score > 75th percentile	4.91	1.30–18.5	0.02

PHQ-9: patient health questionnaire-9.

## Data Availability

The data that support the findings of this study are available from the corresponding author upon request.

## Supplementary Materials

The following supporting information can be downloaded at: https://www.mdpi.com/article/10.3390/ijerph19031899/s1, Table S1: Baseline hematologic, biochemical, inflammatory, and lipid markers of the study population.; Table S2: Laboratory characteristics of the study population stratified by the presence of self-reported chronic pain; Table S3: Clinical, psychometric, and laboratory characteristics of patients with impaired renal function (eGFR < 60 mL/min/1.73 m^2^) stratified by self-reported chronic pain.
