# Supplementary material for: Chronic Pain and Its Association with Depressive Symptoms and Renal Function in Hypertensive Patients
Source: Int J Environ Res Public Health. 2022 Feb 8;19(3):1899. doi: 10.3390/ijerph19031899 (PMC8835698; doi:10.3390/ijerph19031899)
Supplement: Supplementary file 1 [file ijerph-19-01899-s001.zip › ijerph-1552728-supplementary.pdf]

Contents

Supplementary Table 1 2

Supplementary Table 2 3

Supplementary Table 3 4

Supplementary Table 1

**Baseline hematologic, biochemical, inflammatory, and lipid markers of the study population.**				
**Parameters**	**Total Patients (N=158)**	**Patients with eGFR<60 (N=47)**	**Patients with eGFR≥60 (N=111)**	**p**
Ht, %	41.8 (SD: 4.0)	41.3 (SD: 4.4)	41.9 (SD: 3.9)	.52
Hb, g/dl	14.0 (SD: 1.5)	13.9 (SD: 1.7)	14.0 (SD: 1.5)	.71
Glucose, mg/dl	104 (SD: 29)	116 (SD: 45)	99 (SD: 17)	.001
Urea, mg/dl	45 (SD: 25)	61 (SD: 29)	38 (SD: 21)	<.001
Creatinine, mg/dl	1.23 (SD: 0.87)	1.70 (SD: 0.61)	1.03 (SD: 0.89)	<.001
$eGFR_{CKD\text{-}EPI}$, $ml/min/1.73m^2$	70.4 (SD: 22.1)	42.9 (SD: 11.0)	82.1 (SD: 13.7)	<.001
Total cholesterol, mg/dl	204 (SD: 40)	193 (SD: 41)	208 (SD: 39)	.09
Triglycerides, mg/dl	126 (SD: 57)	130 (SD: 70)	125 (SD: 53)	.68
High density lipoprotein cholesterol, mg/dl	57 (SD: 16)	54 (SD: 18)	58 (SD: 15)	.24
Low density lipoprotein, mg/dl	122 (SD: 35)	113 (SD: 34)	125 (SD: 34)	.15
Serum albumin, g/dl	4.3 (SD: 0.7)	4.2 (SD: 0.6)	4.3 (SD: 0.7)	.41
C-reactive protein, mg/l	6.2 (SD: 2.1)	7.0 (SD: 3.0)	6.0 (SD: 2.6)	.84
Fibrinogen, mg/dl	360 (SD:112)	372 (SD: 173)	356 (SD: 86)	.67
SD: standard deviation, Ht: hematocrit, Hb: hemoglobin, eGFR: estimated glomerular filtration rate				

Supplementary Table 2

**Supplementary table 2. Laboratory characteristics of the study population stratified by the presence of self-reported chronic pain.**			
**Parameters**	**Self-reported chronic pain (N=70)**	**No self-reported chronic pain (N=88)**	**p**
Ht, %	41.1 (SD: 3.6)	42.3 (SD: 4.2)	.08
Hb, g/dl	13.6 (SD: 1.3)	14.3 (SD: 1.7)	.02
Glucose, mg/dl	107 (SD: 35)	102 (SD: 23)	.29
Urea, mg/dl	46 (SD: 23)	44 (SD: 27)	.64
Creatinine, mg/dl	1.16 (SD: 0.51)	1.28 (SD: 1.09)	.37
$eGFR_{CKD-EPI}$, ml/min/1.73m^2	67.0 (SD: 21.8)	73.1 (SD: 22.1)	.09
Total cholesterol, mg/dl	208 (SD: 47)	201 (SD: 34)	.37
Triglycerides, mg/dl	131 (SD: 67)	122 (SD: 48)	.47
High density lipoprotein cholesterol, mg/dl	58 (SD: 17)	56 (SD: 15)	.59
Low density lipoprotein, mg/dl	124 (SD: 40)	120 (SD: 31)	.57
Serum albumin, g/dl	4.2 (SD: 0.7)	4.4 (SD: 0.7)	.20
C-reactive protein, mg/l	4.7 (SD: 1.5)	7.7 (SD: 3.8)	.47
Fibrinogen, mg/dl	334 (SD: 72)	376 (SD: 129)	.23
SD: standard deviation, Ht: hematocrit, Hb: hemoglobin, eGFR: estimated glomerular filtration rate			

Supplementary Table 3

Supplementary Table 3: Clinical, psychometric, and laboratory characteristics of patients with impaired renal function (eGFR<60ml/min/1.73m^2) stratified by self-reported chronic pain.			
**Parameters**	**Self-reported pain (N=23)**	**No self-reported pain (N=24)**	**p**
**Clinical characteristics**			
Age, years	63.4 (SD: 12.1)	53.3 (SD: 13.9)	.01
Male Sex	9 (39.1%)	18 (75.0%)	.01
BMI, kg/m^2	28.4 (SD: 3.7)	27.9 (SD: 4.1)	.74
Obesity	5 (29.4%)	6 (33.3%)	.80
Diabetes mellitus	6 (28.6%)	2 (10.0%)	.13
Cardiovascular disease	0	4 (20.0%)	.03
**Psychometric characteristics**			
Depression (PHQ-9)	9.1 (SD: 5.7)	4.0 (SD: 3.0)	<.001
PHQ-9 above 75th percentile	12 (50.0%)	3 (13.0%)	.007
**Laboratory measurements**			
Ht, %	40.0 (SD: 4.0)	42.6 (SD: 4.5)	.10
Hb, g/dl	13.3 (SD: 1.4)	14.5 (SD: 1.8)	.05
Glucose, mg/dl	119 (SD: 51	113 (SD: 38)	.66
Total cholesterol, mg/dl	206 (SD: 55)	183 (SD: 25)	.16
Triglycerides, mg/dl	141 (SD: 100)	123 (SD: 39)	.53
High density lipoprotein cholesterol, mg/dl	56 (SD: 23)	52 (SD: 13)	.51
Low density lipoprotein, mg/dl	122 (SD: 45)	107 (SD: 24)	.29
Serum albumin, g/dl	3.9 (SD: 0.7)	4.4 (SD: 0.4)	.02
C-reactive protein, mg/l	9.8 (SD: 6.5)	4.7 (SD: 1.8)	.43
Fibrinogen, mg/dl	298 (SD: 43)	415 (SD: 207)	.30
ST: standard deviation, eGFR: estimated glomerular filtration rate, BMI: body mass index, PHQ-9: patient health questionnaire-9, Ht: hematocrit, Hb: hemoglobin			
